# Unraveling the Anti‐Tumor Effects and Molecular Mechanisms of Hairyvein Agrimonia Herb in Gastric Cancer Through Network Pharmacology and Experimental Validation

**DOI:** 10.1002/cnr2.70169

**Published:** 2025-05-20

**Authors:** Hequn He, Xiaohui Jin, Xiaoyun Ding, Haizhong Jiang, Xuguang Wang, Yi Chen, Jiyun Zhu

**Affiliations:** ^1^ Emergency Department The First Hospital of Haishu District Ningbo City; ^2^ Gastroenterology Department The First Affiliated Hospital of Ningbo University Ningbo China; ^3^ Hepatopancreatobiliary Surgery Department The First Affiliated Hospital of Ningbo University Ningbo China

**Keywords:** gastric cancer, Hairyvein Agrimonia Herb, pharmacochemistry network

## Abstract

**Background:**

Stomach cancer has become one of the most common types of cancer, with its mortality rate ranking third in the world. Currently, the main treatments for gastric cancer are surgery, radiation therapy, and chemotherapy. Although current treatments can effectively prevent postoperative metastasis and recurrence of gastric cancer, they may also bring various adverse reactions in the gastrointestinal tract and side effects such as bone marrow suppression. Years of research have confirmed that traditional Chinese medicine treatment for gastric and other cancers has distinct characteristics and advantages. Combined treatment can increase the tumor inhibition rate, reduce the side effects of radiation and chemotherapy, improve patients' quality of life, and prolong the survival prognosis.

**Aims:**

This study explores the anti‐tumor effect and specific molecular mechanism of Hairyvein Agrimonia Herb on gastric cancer.

**Methods and Results:**

The combination of network pharmacology technology and various experimental techniques, including in vitro cell verification, was carried out throughout the whole study. The results show that five active components in the Hairyvein Agrimonia Herb can act on 160 carcinogenic targets in gastric cancer. String correlation analysis, Cytoscape network topology analysis, core target screening, protein molecule docking, immunohistochemical expression levels, and survival immune correlation analysis revealed that the carcinogenic genes JUN, HIF1A, and PTGS2 may be the primary drug targets for Hairyvein Agrimonia Herb in treating gastric cancer. The active component quercetin shows the best inhibitory effect on the docking of the PTGS2 protein. Furthermore, the prognostic models constructed by the carcinogenic genes JUN, HIF1A, and PTGS2 are significantly correlated with the survival time of gastric cancer patients.

**Conclusion:**

This study provides a new ethical basis and research direction for understanding the mechanism of action of Hairyvein Agrimonia Herb in treating gastric cancer.

## 1 Introduction

Gastric carcinoma (GC), a malignant neoplasm originating from the epithelium of the gastric mucosa, stands as a prevailing challenge within the realm of gastroenterology. Manifesting with clinical manifestations such as upper abdominal pain, discomfort, bloating, and weight loss, this malignancy poses a significant threat to human life and health on a global scale [1, 2]. Epidemiological data underscore the prominence of gastric cancer as the fourth most commonly diagnosed gastrointestinal malignancy worldwide, with the third‐highest mortality rate, indicative of its profound impact [1, 2]. Within the borders of China alone, an alarming 679,000 new cases of gastric cancer emerge annually, leading to approximately 498,000 deaths, painting a grim picture of the disease burden in the region [3]. Present treatment modalities encompass surgical interventions, radiotherapy, chemotherapy, and molecular‐targeted therapies; however, their efficacy in enhancing overall survival (OS) rates remains limited [4]. Compounding the challenge, the elusive nature of gastric cancer onset often results in diagnoses at advanced stages, curtailing the opportunity for optimal therapeutic interventions and yielding discouragingly low 5‐year survival rates. Hence, the quest for novel treatment avenues capable of ameliorating the prognostic outcomes for gastric cancer patients persists as a pivotal agenda in contemporary gastric cancer research.

Hairyvein Agrimonia (Xianhecao), also known as dragon sprout grass, strength‐draining grass, wolf's teeth grass, etc., is the dried aerial part of Agrimonia pilosa from the Rosaceae family. It was first mentioned in “Huainan Herbal,” characterized by its slightly sweet and neutral nature, with a bitter and astringent taste, and it is associated with the lung, liver, spleen, and large intestine meridians, having the effects of astringing to stop bleeding and tonifying to strengthen the body. Modern pharmacological research has found that Hairyvein Agrimonia possesses functions such as hemostasis, activation of blood circulation, anti‐inflammation, anti‐oxidation, anti‐tumor, and blood sugar regulation [5, 6, 7]. A review of the clinical application and experimental research on Hairyvein Agrimonia shows that its anti‐tumor effects are mainly achieved through inhibiting cell proliferation and promoting cell apoptosis [8]. However, the molecular mechanisms underlying the effects of Hairyvein Agrimonia still need to be clarified, and analyses are not deep enough, with most studies limited to component analysis and functional verification, lacking in‐depth research on specific mechanisms for inhibiting proliferation and inducing apoptosis.

**FIGURE 1:**
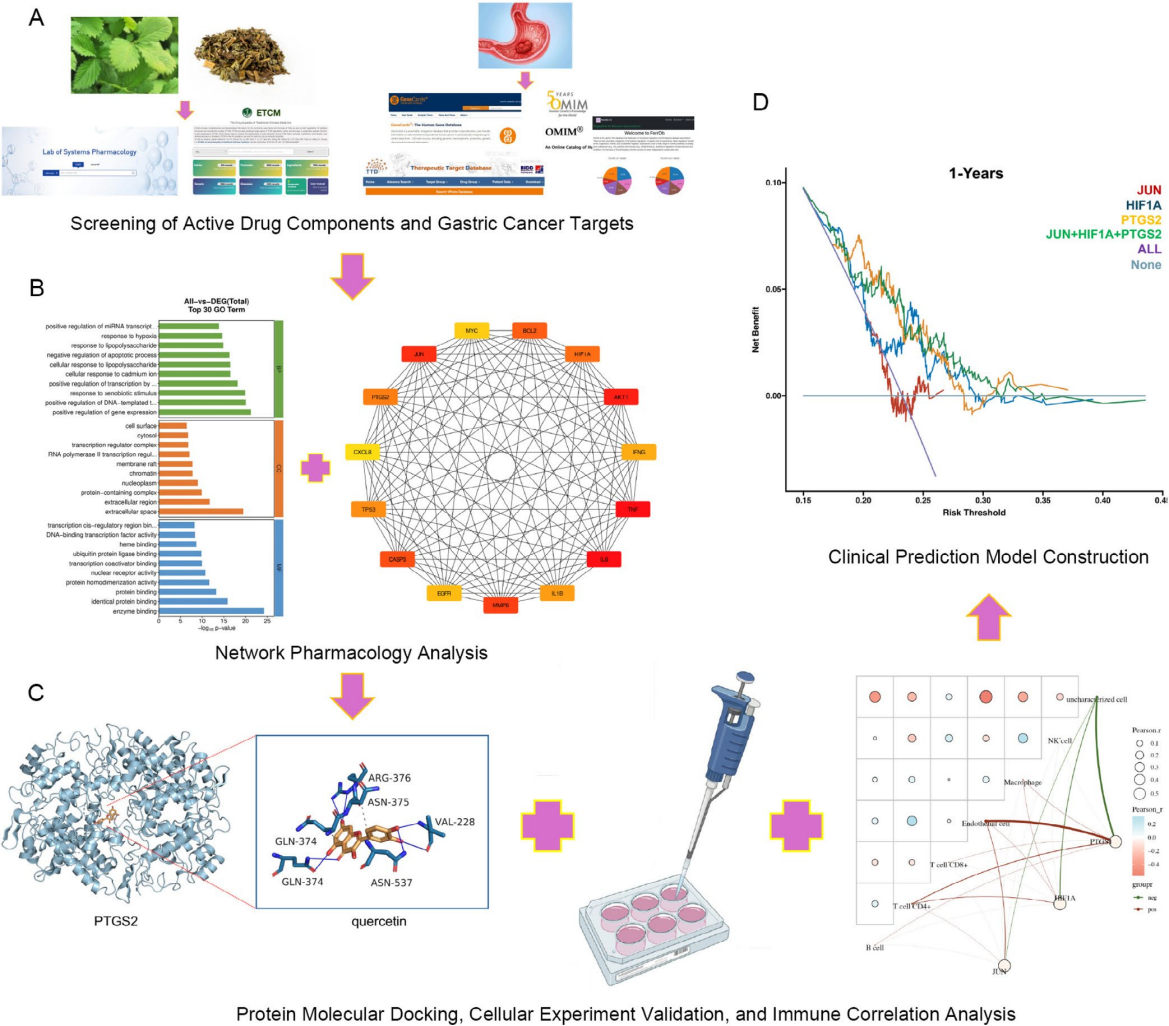
Workflow of the network pharmacological study of Hairyvein Agrimonia in treating gastric cancer (A, B, C, D).

Using network pharmacology docking technology combined with in vitro cell validation experiments, this study screened and explored the main active components of Hairyvein Agrimonia in treating gastric cancer, their corresponding target proteins, and potential signaling pathways. Finally, a prognostic model of the target genes corresponding to Hairyvein Agrimonia was constructed, providing a new theoretical basis and research direction for studying the mechanism of action of the traditional Chinese medicine Hairyvein Agrimonia in treating gastric cancer (Figure 1).

## 2 Materials and Methods

### 2.1 Identification of Potential Targets for Active Constituents in Hairyvein Agrimonia Herb

The active constituents of Hairyvein Agrimonia Herb were systematically retrieved from the TCMSP (https://old.tcmsp‐e.com/tcmsp.php) and ETCM (http://www.tcmip.cn/ETCM/) databases, following stringent screening criteria to select candidates suitable for further investigation. Subsequently, the drug target profiles associated with these selected active components were obtained. To ensure data integrity and consistency, the gene symbols were standardized, and redundant targets pertaining to Hairyvein Agrimonia Herb were meticulously removed utilizing the UniProt ID (https://www.uniprot.org/) platform [9, 10, 11].

### 2.2 Identification of Gastric Cancer Targets

Gastric cancer targets were sourced from prominent databases including Online Mendelian Inheritance in Man (https://omim.org/), DisGeNet (https://www.disgenet.org/home/), the Therapeutic Target Database (https://db.idrblab.net/ttd/), and GeneCards (https://www.genecards.org/home). Notably, the GeneCards database applied a stringent screening threshold of a relevance score of ≥ 10. Subsequent to the removal of redundant entries, a refined list of target genes associated with gastric cancer was delineated. To illuminate potential intersections and synergies, these identified genes were meticulously compared with the target genes of Hairyvein Agrimonia Herb. Through a rigorous curation process aimed at eliminating duplications, the pivotal target genes pertinent to this study were effectively identified [12, 13, 14, 15].

### 2.3 Construction of Protein–Protein Interaction Network

Intersectional target genes derived from the aforementioned datasets were meticulously identified utilizing the innovative capabilities of the OECloud platform (https://cloud.oebiotech.com/#/bio/tools?cat_id&tag_id). Subsequently, a comprehensive Protein–Protein Interaction (PPI) network was intricately crafted on the cutting‐edge STRING platform version 11.0 (https://string‐db.org), leveraging a stringent medium confidence threshold score of 0.400. This network serves as a foundational resource for further investigative endeavors [16, 17].

### 2.4 Drug‐Compound‐Target‐Pathway‐Disease Network

To elucidate the intricate interplay between intersecting target genes, disease entities, Hairyvein Agrimonia Herb, and enrichment analyses, an advanced topological network analysis was orchestrated utilizing the robust capabilities of Cytoscape software version 3.9.0. This comprehensive approach facilitated the construction of a sophisticated Drug‐Compound‐Target‐Pathway‐Disease (DCTPD) network model, encapsulating the dynamic relationships among drugs, active compounds, intersecting genes, and disease states [18].

### 2.5 Functional and Pathway Enrichment Analyses

Leveraging the computational prowess of R software and Bioconductor packages, a comprehensive suite of analytical tools was employed to conduct a rigorous exploration of Gene Ontology (GO), Kyoto Encyclopedia of Genes and Genomes (KEGG), and Reactome pathway enrichment analyses on the enriched target genes derived from the outcomes. Imposing stringent screening criteria of *p* < 0.05 and *q*‐value < 0.05, this analytical framework enabled the discernment of biologically significant functions and pathways intricately linked to the enriched target gene set.

### 2.6 Key Target Gene Identification

Subsequent to the comprehensive enrichment analysis and the utilization of Cytoscape's pivotal target screening algorithm, a refined selection process was initiated to identify key target genes. This iterative approach encompassed a multi‐faceted evaluation of core target genes based on immunohistochemical protein expression levels sourced from the Protein Atlas database (https://www.proteinatlas.org/), survival curve analyses available through the KM Plotter platform (https://kmplot.com/analysis/), and immune‐related assessments facilitated by the TIMER database (http://timer.cistrome.org/) [19, 20, 21].

### 2.7 Molecular Docking Analysis of Compound‐Target Interactions

The 3D molecular structures corresponding to five active components inherent to Hairyvein Agrimonia Herb were procured from the esteemed PubChem database (https://pubchem.ncbi.nlm.nih.gov/). Subsequent to format conversion facilitated by OpenBabel, molecular docking simulations were meticulously executed employing AutoDock Tools version 1.5.7. The ensuing docking outcomes were adeptly scrutinized and visually interpreted utilizing the Protein‐Ligand Interaction Profiler. A stringent screening criterion anchored on a docking score threshold of < −4.25 kcal/mol was applied to discern and prioritize promising compound‐target interactions [22, 23, 24, 25].

### 2.8 Cell Culture

Human ovarian cell lines AGS and HGC‐27 were meticulously cultured in F12K and 1640 media supplemented with 10% fetal bovine serum (FBS) sourced from Gibco, USA. The authenticity of the cell lines was ensured through DNA fingerprinting analysis post procurement from Pricella. Standardized cell culture protocols were meticulously adhered to, with all cell lines incubated under optimal conditions at 37°C and 5% CO_2_. Rigorous passaging practices were implemented to maintain cell viability, with passage intervals strictly limited to < 2 months following initial resuscitation.

### 2.9 Antibodies and Drug

The following primary antibodies were used in this study: PTGS2 (AF7003) and GAPDH (AF7021) (Affinity, China). Luteolin, quercetin, kaempferol, and (+)‐catechin were obtained from the MCE (Shanghai, China).

### 2.10 Colony Formation Assay

In triplicate, cells were meticulously seeded at a density of 2000 cells per well in 6‐well plates and allowed to proliferate over an 8‐day period. Subsequent to incubation, cells were rigorously washed thrice with phosphate buffered saline (PBS), fixed with methanol for a duration of 30 min, and stained with crystal violet at room temperature for an additional 30 min. Following staining, excess dye was thoroughly rinsed off with distilled water. Photographic documentation of the plates was conducted, and quantitative analysis of the colony numbers was undertaken, with statistical evaluation performed using established methodologies.

### 2.11 MTT Assay

Cells were meticulously seeded in a 96‐well plate at a density of 1 × 10^4^ cells per well, with each sample exhibiting triplicate replicates to ensure data robustness. Following a 24‐h incubation period post‐drug treatment initiation, the culture medium was replenished at 48‐h intervals. Assessment of cell viability was conducted utilizing the MTT assay protocol. Briefly, cells were subjected to a 30‐min incubation with 50 μL of 0.2% MTT solution at 37°C within a 5% CO_2_ incubator. Subsequently, the absorbance at 560 nm was quantified using a 96‐well plate reader (Dynex Technologies), with the resultant data subjected to rigorous correlation analysis to derive meaningful insights.

### 2.12 Flow Cytometry Analysis

Following a 24‐h incubation period post‐drug treatment initiation, cells were meticulously cultured to attain optimal conditions for experimental evaluation. Subsequently, the harvested cells were subjected to a series of preparatory steps, including washing with PBS and fixation in ice‐cold 70% ethanol overnight. The fixed cell populations were resuspended in PBS supplemented with 0.2 mg/mL RNAse and 10 mM Propidium Iodide (PI), followed by a 30‐min incubation in a light‐protected environment at 37°. Utilizing a FACSCalibur flow cytometer (Becton Dickinson, CA), flow cytometric analysis was performed to discern cellular characteristics, with data interpretation executed through meticulous correlation analyses to unveil significant associations.

### 2.13 Assessment of Invasion Using Trans‐Well Assay

The matrigel invasion chambers (8 μm, 24‐well cell culture inserts) were used for the cell invasion assay, following the instructions provided by the manufacturer. A total of 50,000 cells were suspended in 500 μL of RPMI1640 or F12K and introduced into the upper chamber. In the lower chamber, 500 μL of RPMI1640 or F12K containing 10% FBS were utilized as a chemoattractant. Following a 24‐h incubation period, the cells located on the top side of the membrane were eliminated using a cotton swab, and subsequently, the membranes were stained with crystal violet. Cell numbers were calculated by taking the average of cell counts obtained from nine distinct view fields at either 10× or 20× magnification.

### 2.14 RT‐qPCR and Western Blot Analysis

Quantitative analysis of RNA levels was conducted utilizing RT‐qPCR methodology in strict adherence to the manufacturer's prescribed guidelines. Simultaneously, Western blot analysis was meticulously executed following the manufacturer's stipulated protocol. Upon cell collection, lysis was carried out on ice utilizing a lysis buffer supplemented with phenylmethylsulfonyl fluoride, a phosphatase inhibitor, and a protease inhibitor cocktail to ensure sample integrity. The subsequent Western blot procedure adhered to standard protocols specified in the manufacturer's instructions. Visualization of protein bands was achieved via a chemiluminescence system (Tanon 5200, Tanon, China). Grayscale analysis of the target protein bands and corresponding loading controls was conducted using dedicated software to ascertain the relative protein levels of interest. Subsequently, the calculated ratio was subjected to thorough correlation testing to unveil pertinent associations.

### 2.15 The Human Protein Atlas

The Human Protein Atlas stands as an integrated database encompassing transcriptomics, proteomics, and systems biology analyses, meticulously mapping diverse tissues, organs, and cell types. Within this comprehensive resource, intricate protein expression profiles spanning both tumor and normal tissues are cataloged, offering invaluable insights into cellular functions and molecular interactions. In the context of this research endeavor, immunohistochemical analyses were adeptly performed to elucidate and characterize the expression patterns of the genes under investigation [19].

### 2.16 The Kaplan–Meier Plotter

Distinguished for its prowess in evaluating the association between mRNA, miRNA, protein, and DNA expression levels with the survival outcomes across a spectrum of 21 distinct human cancer types, the Kaplan–Meier Plotter stands as a versatile tool in oncological research. Employing sophisticated statistical methodologies such as Cox proportional hazards regression and false discovery rate calculation, this platform offers robust insights into prognostic markers and survival correlates. Renowned as a global benchmark, the KM‐Plotter serves as an indispensable resource for the discovery and validation of pivotal survival biomarkers [20].

### 2.17 TIMER: A Robust Tool for Immune Cell Infiltration Analysis

At the forefront of immune cell infiltration analysis in diverse cancer types, TIMER stands as a pivotal resource offering a systematic approach to dissecting the intricacies of immune cell populations within tumor microenvironments. Leveraging a spectrum of immune deconvolution methodologies, this web server yields precise quantification of immune cell infiltrates, enabling researchers to glean profound insights into the immune landscape of tumors. Furthermore, TIMER's capacity to generate sophisticated graphical representations empowers investigators to delve into the intricate interplay among immune signatures, clinical parameters, and genomic attributes, fostering a comprehensive understanding of the tumor immune microenvironment [21].

### 2.18 Ethics Approval and Participant Consent

Adhering rigorously to the ethical principles outlined in the Declaration of Helsinki, the study was meticulously conducted with the utmost regard for participant welfare and ethical standards. The bioinformatic analyses utilized in this research endeavor were sourced from the esteemed Ethics Committee of the First Affiliated Hospital of the Medical School of Ningbo University, ensuring the ethical oversight necessary to uphold the integrity and welfare of all individuals involved.

### 2.19 Statistical Analysis

The statistical results are meticulously showcased as means ± standard deviation derived from a minimum of three independent biological replicates, underscoring the robustness of the experimental data. Comparative analyses between two groups were discerned using Student's *t*‐test, while distinctions among multiple groups were evaluated through one‐way analysis of variance (ANOVA), ensuring comprehensive data interpretation. Survival analyses were conducted utilizing the Kaplan–Meier method, with inter‐group comparisons facilitated by the log‐rank test to ascertain noteworthy differences. Spearman's rank correlation analysis was employed to dissect the associations between variable pairs, providing valuable insights into potential relationships. Notably, these analyses were meticulously executed with the aid of SPSS (V20, IBM Corp., Armonk, USA) and the GraphPad Prism 8.0 program, accentuating the methodological rigor underpinning the data interpretation process. The figures indicate statistical significance at *p* < 0.05 (*), *p* < 0.01 (**), *p* < 0.001 (***), and *p* < 0.0001 (****).

## 3 Results

### 3.1 Acquisition and Standardization of Active Components From Hairyvein Agrimonia

The active components inherent to Hairyvein Agrimonia were meticulously sourced through the TCMSP (https://old.tcmsp‐e.com/tcmsp.php) and ETCM (http://www.tcmip.cn/ETCM/) databases, culminating in the identification of five potent constituents meriting further investigation. Subsequently, the pertinent drug target data associated with these select components was diligently retrieved, laying the foundation for subsequent research endeavors. Noteworthy efforts were directed toward standardizing the gene symbols by leveraging the UniProt ID resource (https://www.uniprot.org/), a pivotal step aimed at ensuring data harmonization and integrity. Through this meticulous process, duplicate targets specific to Hairyvein Agrimonia were judiciously identified and eliminated, as outlined in Table 1 for enhanced clarity and reference.

**TABLE 1:** Identification of 6 chemical components absorbed from the TCMSP and ETCM.

Mol ID	Molecule name	MW	AlogP	Hdon	Hacc	OB (%)	Caco‐2	BBB	DL	FASA‐	HL
MOL001002	Ellagic acid	302.2	1.48	4	8	43.06	−0.44	−1.41	0.43	0.43	−1.04
MOL000422	Kaempferol	286.25	1.77	4	6	41.88	0.26	−0.55	0.24	0	14.74
MOL000492	(+)‐catechin	290.29	1.92	5	6	54.83	−0.03	−0.73	0.24	0	0.61
MOL000006	Luteolin	286.25	2.07	4	6	36.16	0.19	−0.84	0.25	0.39	15.94
MOL000098	Quercetin	302.25	1.5	5	7	46.43	0.05	−0.77	0.28	0.38	14.4

### 3.2 Acquisition of Gastric Cancer Targets From Diverse Databases

In a comprehensive effort to delineate the target landscape pertinent to gastric cancer, we meticulously curated data from reputable databases including the Online Mendelian Inheritance in Man (https://omim.org/), DisGeNet (https://www.disgenet.org/home/), the Therapeutic Target Database (https://db.idrblab.net/ttd/), and GeneCards (https://www.genecards.org/home). Notably, the GeneCards database employed a stringent relevance threshold of ≥ 10 to ensure the selection of pertinent targets. Through a systematic curation process and the removal of redundancies, we successfully collated a comprehensive list of target genes associated with gastric cancer. A rigorous cross‐analysis facilitated the identification of 13,898 target genes, meticulously documented for reference and further investigation.

### 3.3 Identification of Intersection Target Genes and PPI Network Construction

**FIGURE 2:**
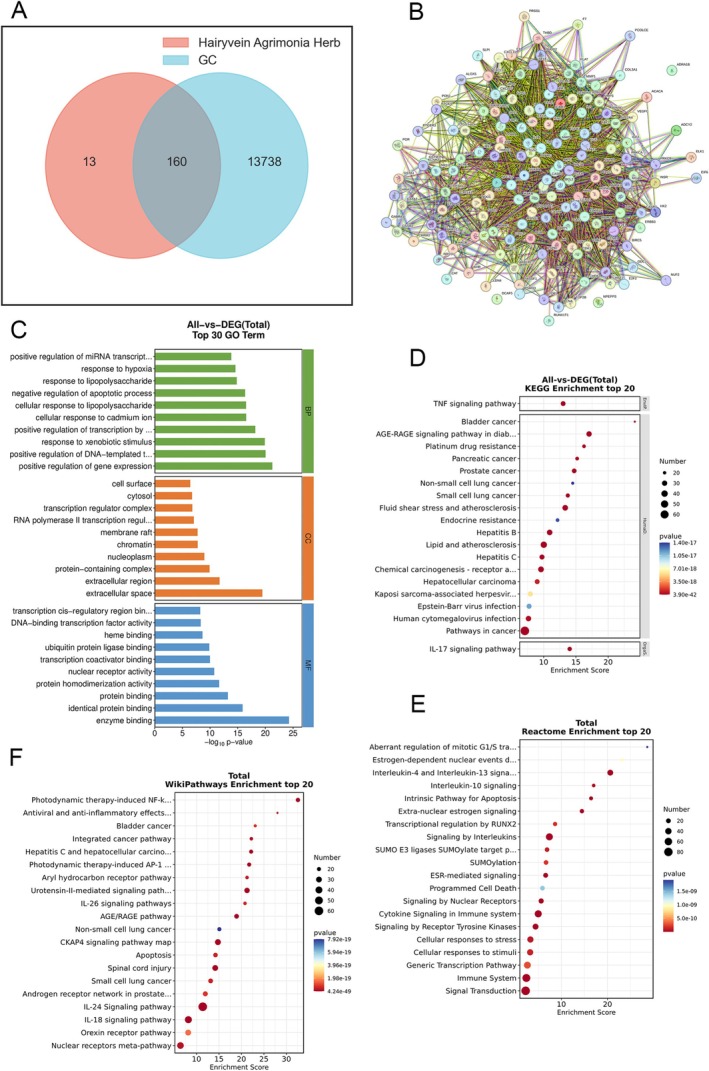
Identification targets of Hairyvein Agrimonia–gastric cancer. (A) Intersection results of Hairyvein Agrimonia drug target genes and gastric cancer target genes. (B) PPI network. The circle nodes represent core targets, and the lines represent different degrees of correlation. (C) GO enrichment analysis. (D) KEGG enrichment analysis. (E) Reactome enrichment analysis. (F) Wiki enrichment analysis.

Utilizing the advanced capabilities of the OECloud platform (https://cloud.oebiotech.com/#/bio/tools?cat_id&tag_id), we meticulously scrutinized the datasets cited earlier, culminating in the discernment of 160 intersecting target genes of significant relevance (depicted in Figure 2A). Subsequently, leveraging the cutting‐edge functionalities of the STRING platform version 11.0 (https://string‐db.org), we embarked on the construction of a protein–protein interaction (PPI) network. Operating at a medium confidence level of 0.400, the PPI network was meticulously delineated to illuminate intricate molecular associations and facilitate forthcoming research endeavors, as visually depicted in Figure 2B.

### 3.4 Functional Analysis of Disease Targets and Pathway Enrichment

Following the identification of disease targets linked to the active components, a comprehensive GO analysis was conducted utilizing the Bioconductor package within the R software environment. The results were meticulously scrutinized to unveil significantly enriched biological processes, subsequently showcased in the accompanying figure. The GO enrichment encompassed biological processes, cellular components, and molecular functions, shedding light on the multifaceted roles of the target genes. Notable associations included cellular response to cadmium ion, RNA polymerase II transcription regulator complex, and transcription coactivator binding, as vividly illustrated in Figure 2C. Expanding the analysis horizon, Kyoto Encyclopedia of Genes and Genomes (KEGG) and WikiPathways enrichment analyses were carried out to unravel the underlying pathways enriched with the identified genes. Remarkably, the outcomes underscored pronounced enrichment in Endocrine and metabolic disease signaling pathways, alongside photodynamic therapy‐induced NF‐κB survival signaling pathways, as delineated in Figure 2D,F, respectively. Further insights were gleaned through Reactome enrichment analysis, unveiling compelling mechanistic pathways potentially underpinning the therapeutic efficacy of Hairyvein Agrimonia in gastric cancer treatment. Noteworthy findings included genes associated with aberrant regulation of mitotic G1/S transition in cancer due to RB1 defects and Estrogen‐dependent nuclear events downstream of ESR‐membrane signaling, hinting at intricate molecular mechanisms contributing to the therapeutic effects, as highlighted in Figure 2E.

### 3.5 Creation of a Comprehensive Network and Core Target Gene Identification

**FIGURE 3:**
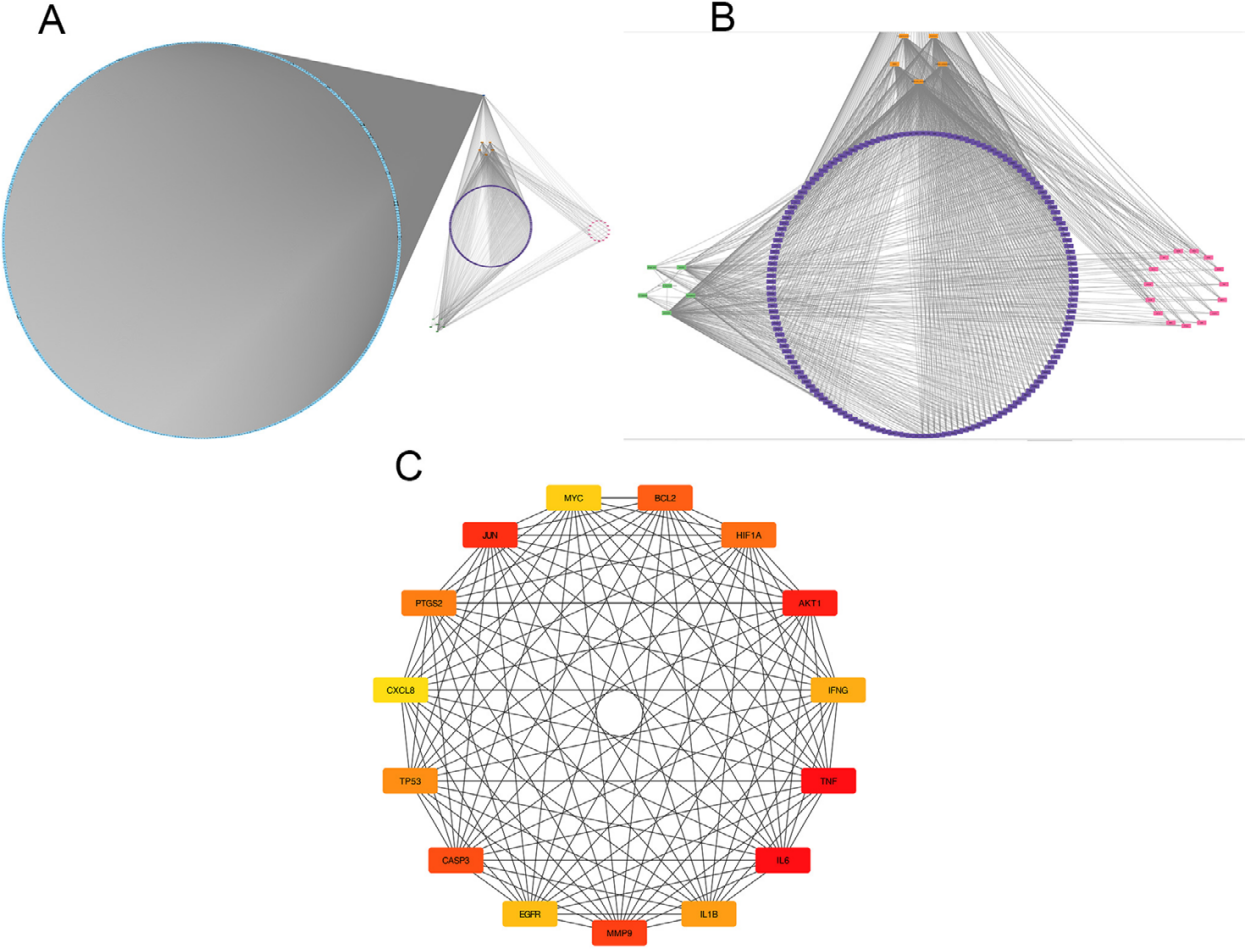
DCTPD network and enrichment analysis. The topological network connections of drugs, active compounds, intersecting genes, signaling pathways, and diseases. (A) DCTPD network. (B) Connections among intersecting genes, active components, and signaling pathways. (C) Linkage results of the top 15 core target genes screened using the Hubba plugin in Cytoscape.

Building upon the aforementioned findings, we meticulously constructed a DCTPD network using Cytoscape version 3.9.0, unveiling the intricate relationships underpinning the therapeutic efficacy of Hairyvein Agrimonia in treating gastric cancer. This network diagram intricately illustrates the complex interplay among diseases, active components, targets, pathways, and drugs, with nodes distinguished by diverse colors and shapes to signify their respective categories. The number of connections linked to each node serves as a gauge of its significance within the network, offering valuable insights into the critical elements shaping the therapeutic landscape. Notably, the network analysis revealed that Hairyvein Agrimonia exhibits connectivity to five potential targets, indicative of the multi‐faceted interactions wherein a single active component may modulate multiple targets, underscoring the nuanced intervention effects of this herbal remedy. Moreover, leveraging the Hubba plugin, we identified the top 15 core target genes pivotal to the therapeutic action of Hairyvein Agrimonia in gastric cancer treatment. This strategic approach provided a focused insight into the key molecular players driving the observed pharmacological effects, as depicted in Figure 3C. Collectively, these analyses offer a comprehensive perspective on the intricate network of interactions and core target genes crucial for delineating the mechanisms underlying Hairyvein Agrimonia's therapeutic potential in combating gastric cancer.

### 3.6 Secondary Screening of Core Target Genes

**FIGURE 4:**
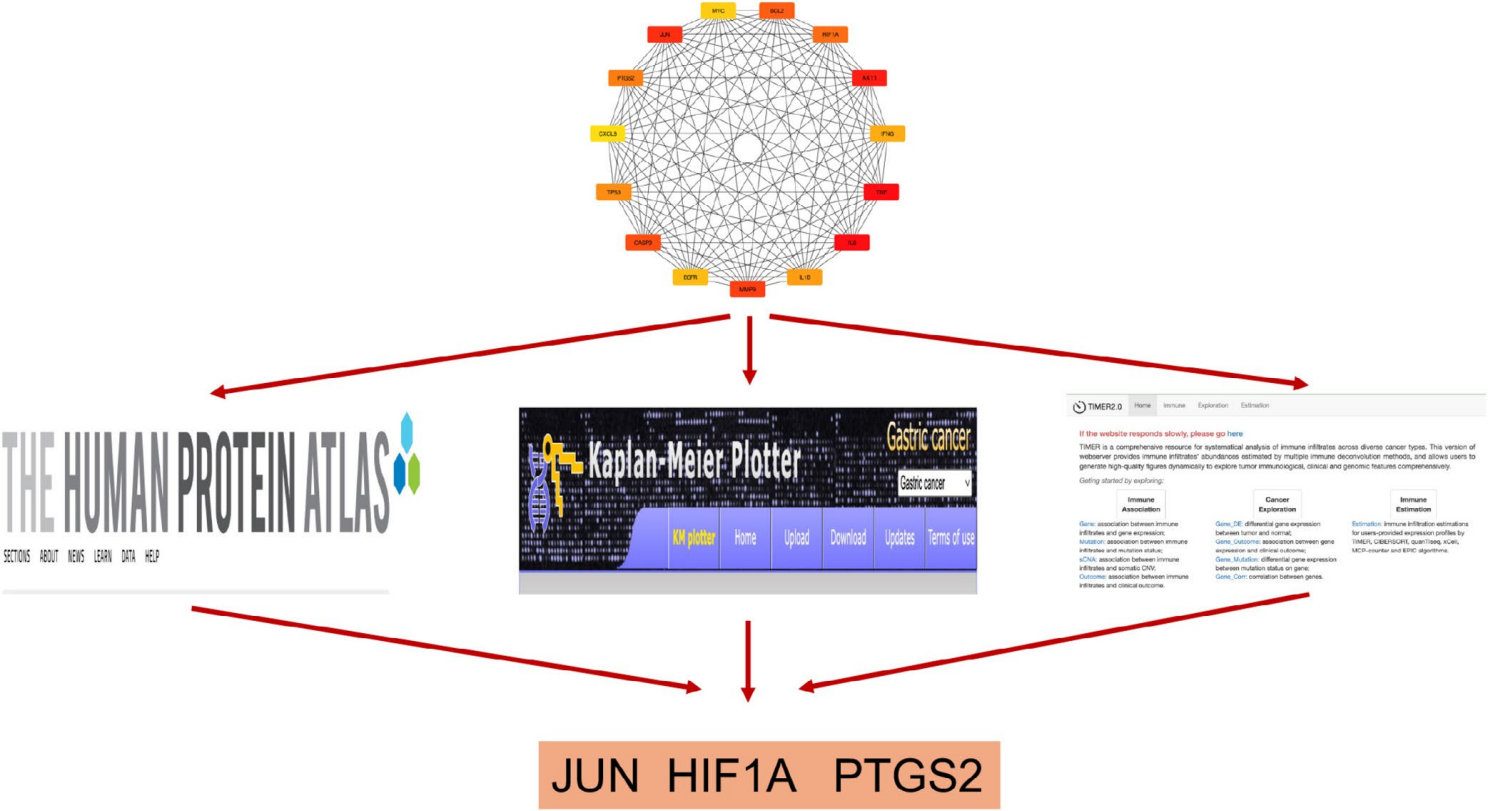
Secondary screening flowchart for 15 core genes.

Subsequently, leveraging the insights gleaned from the identified 15 core target genes, we delved into a comprehensive analysis encompassing the expression profiles of these pivotal targets at the immunohistochemical protein level, exploration of OS curves, and scrutiny through advanced immune infiltration algorithmic methodologies. The culmination of these analyses uncovered compelling correlations, highlighting the intricate interplay between protein expression levels, OS durations, and the extent of immune infiltration pertaining to three notable target genes‐JUN, HIF1A, and PTGS2. Notably, the findings underscored the profound significance of these target genes in shaping the prognosis of individuals afflicted with gastric cancer, as evidenced by statistically significant associations (*p* < 0.05), as depicted in Figure 4.

### 3.7 Analysis of Molecular Docking Results

**FIGURE 5:**
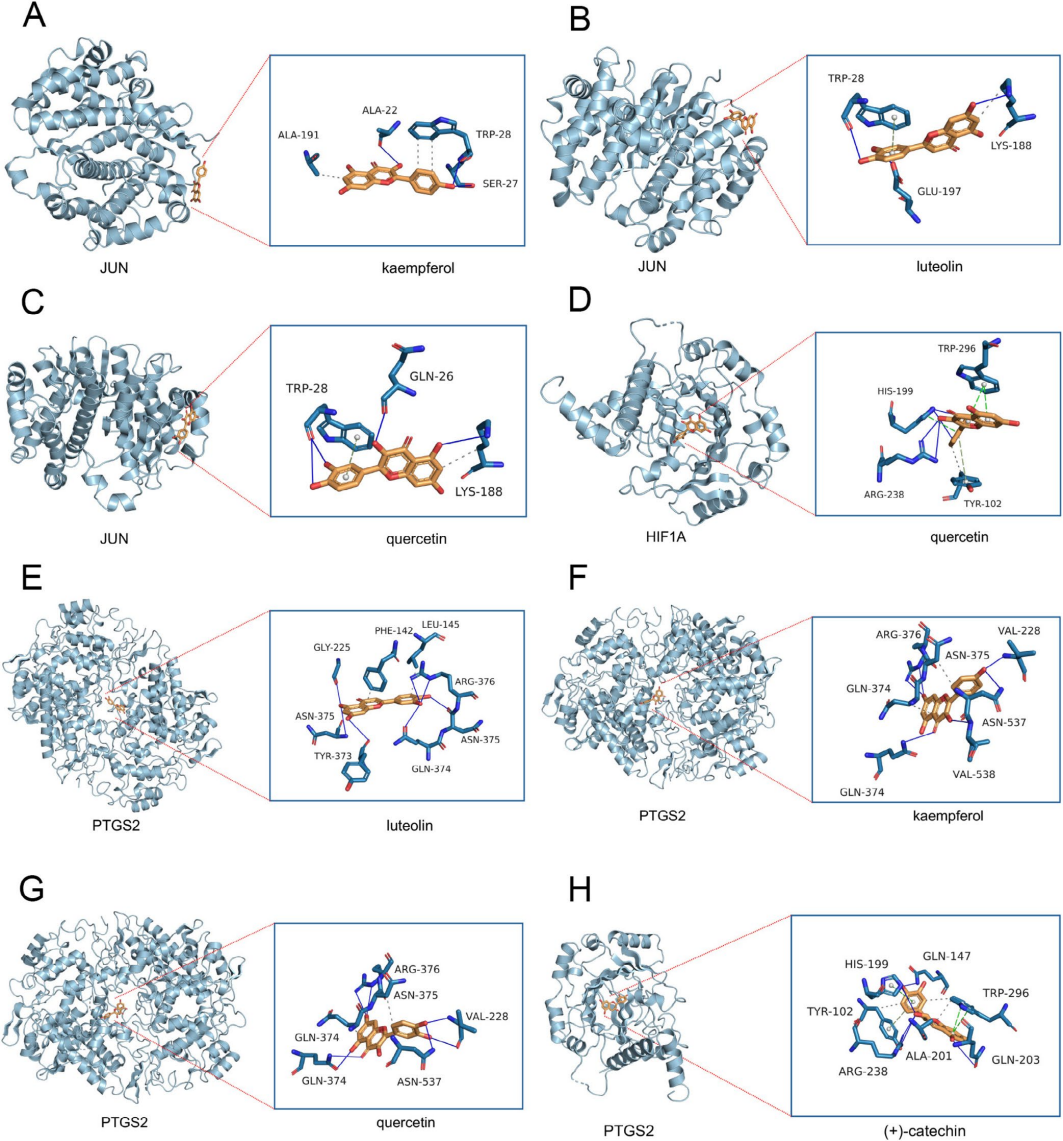
3D docking patterns of key targets and specific active compounds. (A) Kaempferol binding to JUN crystal structure. (B) Luteolin binding to JUN crystal structure. (C) Quercetin binding to JUN crystal structure. (D) Quercetin binding to HIF1A crystal structure. (E) Luteolin binding to PTGS2 crystal structure. (F) Kaempferol binding to PTGS2 crystal structure. (G) Quercetin binding to PTGS2 crystal structure. (H) (+)‐Catechin binding to PTGS2 crystal structure.

In a bid to elucidate the potential therapeutic functionalities of the five active components present in Hairyvein Agrimonia and validate the outcomes derived from network pharmacology analyses, we embarked on molecular docking endeavors aimed at predicting the interactions between these active constituents and three pivotal target genes—JUN, HIF1A, and PTGS2. Employing the state‐of‐the‐art AutoDock software, we meticulously evaluated the protein‐molecule docking complexes, calculating docking affinity scores to gauge the strength of interactions. Notably, docking scores below −4.25 kcal/mol were deemed indicative of favorable docking affinities, as expounded in Table 2. Comprehensive details regarding the docking scores are succinctly delineated in the aforementioned table for reference and scrutiny. Moreover, to enhance the visual representation of these intricate molecular interactions, we meticulously generated 3D structural depictions of the docking complexes, offering a vivid insight into the binding modalities between the target proteins and active components (Figure 5A–H). Noteworthy among the findings was the exemplary docking affinity observed between the PTGS2 protein and quercetin, evidenced by a remarkable binding free energy of −8.9 kcal/mol. This interaction was underscored by hydrophobic interactions with key residues such as ARG‐376, ASN‐375, GLN‐374, VAL‐228, and ASN‐537, as vividly illustrated in Figure 5G. Collectively, these findings shed light on the potential efficacy of Hairyvein Agrimonia in selectively targeting pathological sites implicated in gastric cancer treatment. However, while these computational predictions offer valuable insights, rigorous experimental validation is imperative to substantiate and translate these in silico findings into tangible therapeutic applications.

**TABLE 2:** Target molecule docking results for candidate active components.

Affinity (kcal/mol)				
Compound name	Luteolin	Quercetin	Kaempferol	(+)‐Catechin
JUN	−6.5	−6.7	−6.3	—
HIF1A	—	−7.6	—	—
PTGS2	−8.7	**−8.9**	−8.7	−7.7

### 3.8 In Vitro Validation Experiments on the Inhibition of GC Cells by Active Components of Hairyvein Agrimonia

**FIGURE 6:**
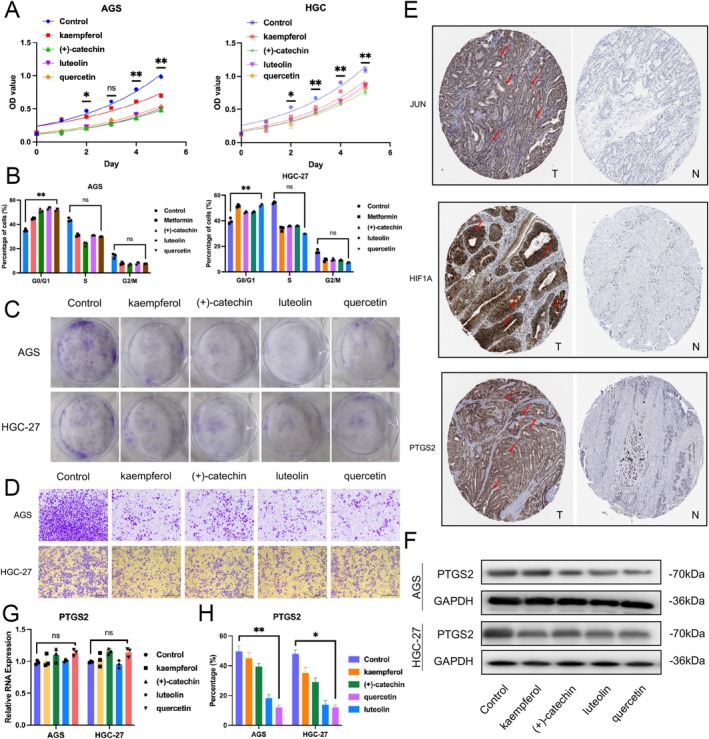
In vitro experiments validating the inhibition of gastric cancer cells by Hairyvein Agrimonia (A) Proliferation rates of Control and luteolin, quercetin, kaempferol, and (+)‐catechin, AGS, and HGC‐27 cells treated as indicated were measured by MTT assay. (B) Flow cytometry analyzed each group cell's cell cycle distribution and treated as indicated. (C, D) After 10 days, cells were stained with crystal violet and imaged to assess their colony formation ability and invasion ability. (E) Immunohistochemistry data from public protein databases show high JUN, HIF1A, and PTGS2 expression in gastric cancer tissues. (G) The PCR results of PTGS2 in AGS and HGC‐27 cells were treated as indicated. (F, H) The western blot results of PTGS2 in AGS and HGC‐27 cells were treated as indicated.

Following the initial confirmation that the active components of Hairyvein Agrimonia had a sound docking basis with the gastric cancer target protein PTGS2, we further carried out in vitro validation experiments using gastric cancer cells. We assessed the inhibitory effects of four active components of Hairyvein Agrimonia (luteolin, quercetin, kaempferol, and (+)‐catechin) on gastric cancer cells through growth curves, colony formation, cell cycle, immunohistochemistry, PCR, and Western blot analyses. The results showed that after 24 h of treatment with the four Hairyvein Agrimonia active components, the growth rate of gastric cancer cells AGS and HGC‐27 was significantly slowed after 2 days (Figure 6A). Similarly, the results of cell cycle and colony formation experiments suggested that after treatment with Hairyvein Agrimonia, gastric cancer cells primarily remained in the G0/G1 phase, with a relative reduction in the S and G2/M phases compared to the control group (Figure 6B), and could significantly inhibit the colony‐forming and invasion ability of AGS and HGC‐27 (Figure 6C,D). Finally, analysis from the immunohistochemistry database revealed that JUN, HIF1A, and PTGS2 were highly expressed in gastric cancer tumor tissues (Figure 6E). Meanwhile, in vitro cell experiment results showed that the four active components could significantly inhibit the protein expression level of PTGS2 without a noticeable suppression of its RNA level (Figure 6F–H). These findings indicate that the active components of Hairyvein Agrimonia can significantly inhibit the growth rate and colony formation ability, slow down the cell cycle progression, and suppress the corresponding protein expression levels of gastric cancer cells AGS and HGC‐27 in vitro.

### 3.9 Analysis of the Correlation Between Hairyvein Agrimonia Target Genes and the Abundance of Immune Cell Infiltration in Gastric Cancer

Upon establishing the robust docking interactions between the active components of Hairyvein Agrimonia and the target protein PTGS2 in gastric cancer, we proceeded with rigorous in vitro validation experiments employing gastric cancer cells. Our comprehensive investigation sought to evaluate the inhibitory effects of four key active components of Hairyvein Agrimonia—luteolin, quercetin, kaempferol, and (+)‐catechin‐on gastric cancer cells via a multifaceted approach encompassing growth curves, colony formation assays, cell cycle analyses, immunohistochemistry, polymerase chain reaction (PCR), and Western blot analyses. The results of our investigations unveiled profound insights into the therapeutic potential of Hairyvein Agrimonia in mitigating gastric cancer progression. Following a 24‐h exposure to the active components, gastric cancer cells AGS and HGC‐27 exhibited a marked deceleration in growth rate, a trend that persisted over the ensuing 2‐day period, as depicted in Figure 6A. Furthermore, cell cycle and colony formation assays underscored a remarkable arrest of the gastric cancer cells in the G0/G1 phase post Hairyvein Agrimonia treatment, accompanied by a relative decline in the S and G2/M phases compared to the control group, as depicted in Figure 6B. Moreover, the inhibitory impact on the colony‐forming ability of AGS and HGC‐27 cells was prominently evident following treatment with Hairyvein Agrimonia, as highlighted in Figure 6C.

**FIGURE 7:**
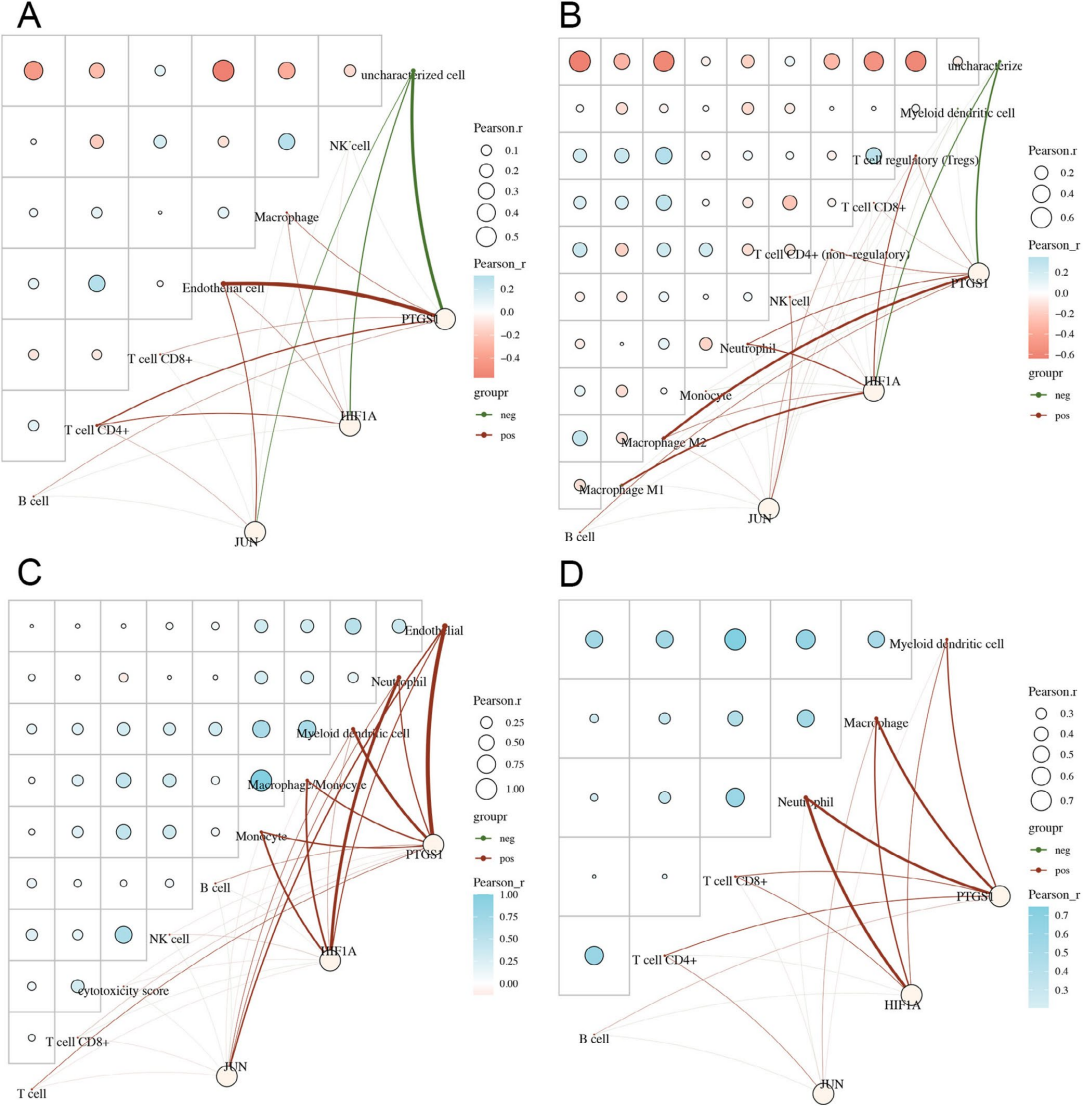
The correlation between JUN, HIF1A, PTGS2, and immune cells in four different immune infiltration algorithms. (A–D) The correlation between the abundance of immune cells and JUN, HIF1A, and PTGS2 expression through EPIC, QUANTISEQ, MCPCOUNTER, and TIMER algorithms. The heatmaps in the figure represent self‐correlation analysis of immune scores, with red indicating positive correlation, blue indicating negative correlation, and the more intense the color, the stronger the correlation. The size of the circle also indicates the strength of the correlation; the schematic shows red lines indicating a negative correlation and green lines indicating a positive correlation between model scores or gene expression and immune scores.

Additionally, immunohistochemical analyses unveiled heightened expressions of JUN, HIF1A, and PTGS2 in gastric cancer tumor tissues (Figure 6D–F), aligning with the in vitro observations. Intriguingly, the in vitro experiments demonstrated a significant reduction in the protein expression levels of PTGS2 following treatment with the active components, despite a subtle impact on its RNA levels, as illustrated in Figures 6G–I and 7. Collectively, these findings underscore the potent inhibitory effects exhibited by the active components of Hairyvein Agrimonia in attenuating the growth rate, colony formation capability, and cell cycle progression, while concomitantly suppressing the protein expression levels of gastric cancer cells AGS and HGC‐27 in an in vitro milieu.

### 3.10 High Expression of JUN, HIF1A, and PTGS2 Genes Associated With Poor Prognosis in Gastric Cancer Patients

**FIGURE 8:**
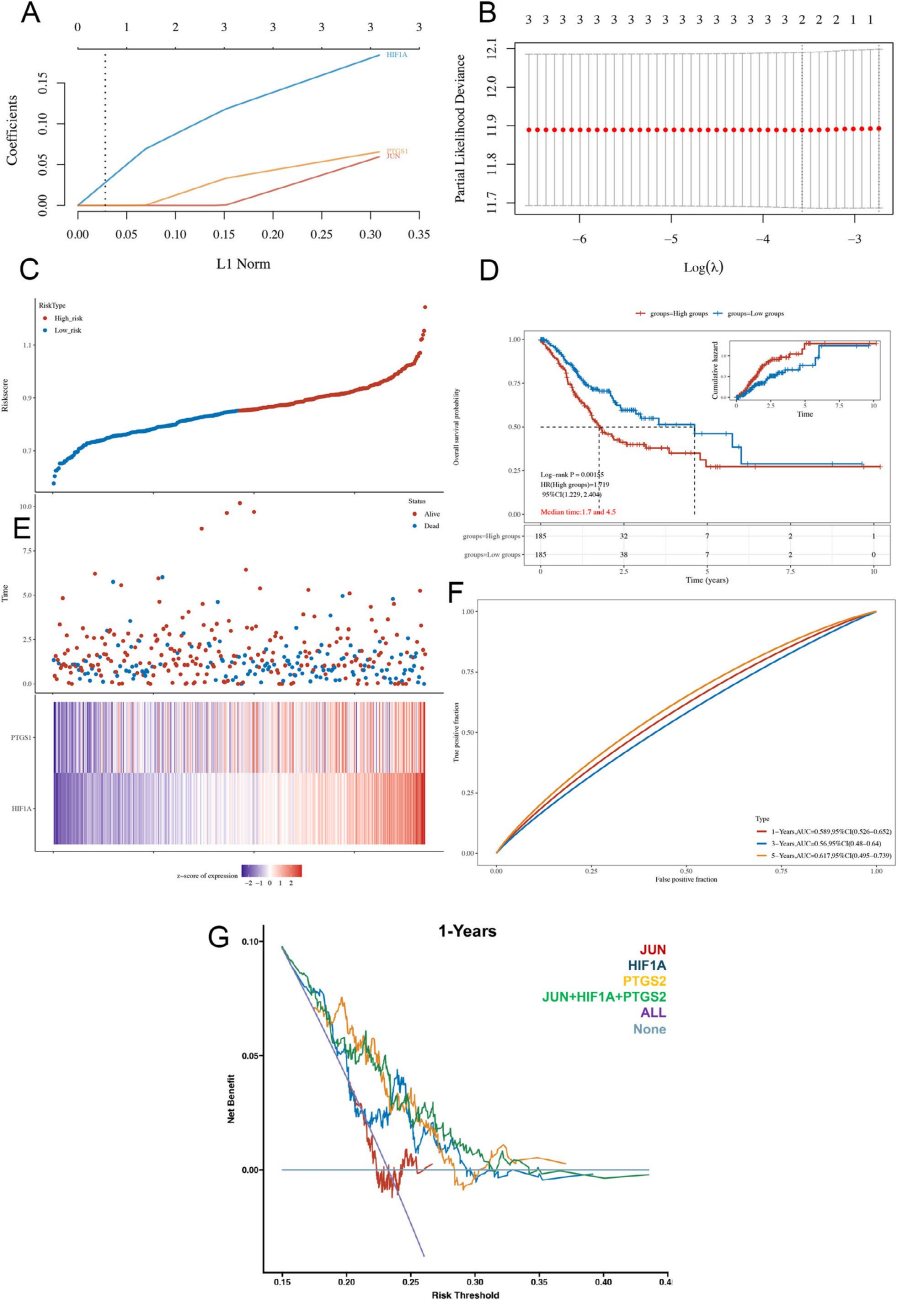
Clinical prognostic potential of three Hairyvein Agrimonia target genes in gastric cancer patients. (A) LASSO coefficients profiles of JUN, HIF1A, and PTGS2 genes. (B) Using the minimum lambda value, LASSO regression with tenfold cross‐validation obtained JUN, HIF1A, and PTGS2 genes. (C) The curve of risk score. (D) Kaplan–Meier survival analysis of the JUN, HIF1A, and PTGS2 genes signature. (E) Survival status of the patients and heatmap of the expression profiles of the JUN, HIF1A, and PTGS2 genes in the low‐ and high‐risk groups. (F) Time‐dependent ROC analysis of the JUN, HIF1A, and PTGS2 genes signature. ROC receiver operating characteristic. (G) JUN, HIF1A, and PTGS2 genes were used to establish the DCA module and analyzed 1‐year OS time.

In the realm of clinical translation, we leveraged the genetic signatures of JUN, HIF1A, and PTGS2 to establish a predictive model tailored for gastric cancer prognosis. Initially, employing the rigors of LASSO regression coupled with 10‐fold cross‐validation techniques, we meticulously determined the optimal lambda value characterized by minimal partial likelihood bias, showcasing a profound association with OS outcomes, as depicted in Figure 8A,B. Subsequently, for each patient, we computed an individualized risk score utilizing the median cut‐off point derived through the “Survminer” R package, effectively stratifying the cohort into high‐risk and low‐risk categories, as illustrated in Figure 8C. Notably, a comprehensive heatmap representation of the four genes further elucidated the intricate molecular landscape underpinning the prognostic model, as showcased in Figure 8E.

Furthermore, Kaplan–Meier (KM) survival analyses unveiled a stark contrast in OS between the high‐risk and low‐risk groups, with median survival durations of 1.7 and 4.5 months, respectively, underscoring the robust prognostic capabilities of the multi‐gene model, as depicted in Figure 8D. Noteworthy advancements were witnessed in the time‐dependent receiver operating characteristic (ROC) analyses, wherein the amalgamation of the three genes exhibited enhanced prognostic efficacy, as evidenced by a substantial area under the curve (AUC) value, indicative of superior predictive power for OS at 1, 3, and 5 years, as visually represented in Figure 8F.

Conclusively, decision curve analyses accentuated the enhanced clinical utility of the combined predictive model incorporating JUN, HIF1A, and PTGS2 genes, surpassing the individual gene prognostic assessments, as delineated in Figure 8G. Collectively, these compelling findings underscore the pivotal role of the JUN, HIF1A, and PTGS2 genes as promising clinical prognostic markers for patients grappling with gastric cancer, heralding a paradigm shift in personalized prognostication strategies within the oncological landscape.

## 4 Discussion

Gastric cancer stands as the fifth most prevalent malignancy globally, ranking as the third leading contributor to cancer‐related mortality. The clinical trajectory for individuals afflicted with gastric cancer remains grim, characterized by a meager 5‐year survival rate ranging from 20% to 30% [26]. Despite notable strides in surgical interventions and anti‐neoplastic drug research spanning the past three decades, the specter of postoperative recurrence and metastasis looms large, perpetuating a dismal scenario marked by stagnant survival rates and compromised quality of life [27]. Against this backdrop, our investigation delves into unraveling the intricate mechanisms underlying the therapeutic actions of active components derived from traditional Chinese medicine (TCM) in combating gastric cancer. By shedding light on the nuanced interplay between these herbal constituents and gastric cancer pathogenesis, our study aspires to chart new vistas in clinical gastric cancer management, heralding a paradigm shift through the exploration of novel herbal components with transformative therapeutic potential.

Traditional Chinese medicine (TCM) considers that a variety of factors act together on the body, leading to organ deficiency, which is a prerequisite for the onset of tumors. Patients with tumors often suffer from deficiency of Qi and blood, Qi stagnation, and blood stasis. At the same time, Hairyvein Agrimonia can replenish deficiency, support health, disperse blood stasis, and dissipate masses [28]. As early as the Ming dynasty, medical experts used Hairyvein Agrimonia to treat esophageal cancer, gastric cancer, and other diseases. Modern research has found that Hairyvein Agrimonia mainly inhibits tumor cell proliferation and induces apoptosis, improving patient symptoms and reducing the adverse effects of chemotherapy, thus playing a role in cancer inhibition, detoxification, and efficacy enhancement. Therefore, Hairyvein Agrimonia, with its efficacy in “inhibiting cancer,” “reducing toxicity,” and “supporting the healthy,” can become one of the choices for anti‐tumor treatment. Clinically, compound formulas mainly containing Hairyvein Agrimonia have specific anti‐tumor effects on lung, gastric, intestinal, liver, and early breast cancer [29].

Hairyvein Agrimonia is also widely used in the treatment of gastrointestinal tumors. Numerous clinical studies on treating gastrointestinal tumors have found that formulas containing Hairyvein Agrimonia inhibit tumor cell proliferation, regulate immune function, and improve hemorheological parameters. Yanglan et al. used the Cancer Detoxification III formula (Hedyotis et al. ternata, *Codonopsis pilosula* , etc.) combined with chemotherapy (SOX protocol) to treat stage IV gastric cancer, significantly improving patient immunity, quality of life, and Karnofsky score, and alleviating clinical symptoms [30]. Ting et al. used Si Ni San combined with Phlegm and Stasis Removal Decoction (Tangerine Peel, *Pinellia ternata* , Poria cocos, *Curcuma zedoaria* , Fried Coix seed, *Gallus gallus* domesticus, Bupleurum chinense, Hedyotis diffusa, Hairyvein Agrimonia, etc.) to treat patients with gastritis and precancerous lesions of gastric cancer, significantly improving changes in gastric mucosal gland atrophy, intestinal metaplasia, increasing the *Helicobacter pylori* eradication rate, and reducing bile reflux [31]. Gao Mengjie used Sha Shen Shi Hu Soup (Radix Adenophorae, Polygala japonica, Fried grain sprout, Hairyvein Agrimonia, Fried Atractylodes macrocephala, Dendrobium nobile, etc.) combined with chemotherapy (S1 monotherapy, XELOX protocol, 4 weeks per cycle, 2 weeks of treatment) in the treatment of 40 patients with advanced gastric cancer. The results showed that the combined medication group significantly improved traditional Chinese medicine symptoms and KPS score and had a particular effect in alleviating the toxic side effects of chemotherapy [32]. This study, through network pharmacology and protein‐molecule docking technology, screened and discovered that luteolin, quercetin, kaempferol, and (+)‐catechin in Hairyvein Agrimonia can inhibit the gastric cancer cell lines AGS and HGC‐27, and the main targets of these active components include oncogenic proteins such as JUN, HIF1A, and PTGS2.

Prostaglandin‐endoperoxide synthase 2 (PTGS2), commonly recognized as cyclooxygenase 2 (COX‐2), serves as the pivotal enzymatic catalyst governing the biosynthesis of prostaglandins (PGs), exerting control over crucial physiological processes [33]. Positioned predominantly within the nuclear membrane, the COX‐2 gene orchestrates the intracellular translocation of PG products, thereby modulating the transcriptional activity of target genes and influencing cellular responses [34]. Within the context of the inflammatory milieu characteristic of gastrointestinal malignancies, PTGS2 assumes a central role, steering a cascade of molecular events with profound implications for tumorigenesis. A wealth of scientific investigations has underscored the impact of heightened PTGS2 expression on the oncogenic landscape, elucidating its regulatory roles in angiogenesis, apoptosis, and cytokine signaling pathways, thereby intricately shaping the trajectory of gastrointestinal carcinogenesis [35]. Noteworthy findings from diverse studies have underscored the aberrant upregulation of PTGS2 in human gastric cancer tissues, highlighting its strategic involvement in disease pathogenesis. Moreover, compelling evidence from experimental models has underscored the therapeutic potential of selective PTGS2 inhibitors such as celecoxib in impeding gastric tumor development, offering promising avenues for targeted intervention strategies in the realm of gastric cancer therapeutics [36].

Moreover, emerging literature has unveiled compelling insights into the intricate interplay between genetic alterations in PTGS2 and microsomal prostaglandin E synthase, showcasing their potential to instigate tumorigenesis within the gastric milieu in transgenic mouse models. Noteworthy experimental evidence has underscored the chemopreventive efficacy of celecoxib in curtailing chemically induced gastric cancer progression in rodent models. Intriguingly, clinical investigations have shed light on the prognostic implications of PTGS2 protein expression patterns in gastric cancer patients, underscoring its association with distinct clinicopathological features including histological subtype, tumor location, size, and lymph node involvement. Notably, the aberrant overexpression of PTGS2 protein emerges as an independent prognostic determinant portending unfavorable survival outcomes [37]. In alignment with these seminal findings, the current study illuminates the profound inhibitory effects of all four active components within Hairyvein Agrimonia on PTGS2 protein expression levels, offering a mechanistic rationale for the anti‐cancer properties exhibited by this traditional herbal remedy. Intriguingly, the integrative predictive model hinging upon the genetic signatures of JUN, HIF1A, and PTGS2 emerges as a robust prognostic indicator correlating with poorer clinical outcomes in gastric cancer patients. Collectively, these findings underscore the potential therapeutic efficacy of Hairyvein Agrimonia in antagonizing gastric cancer progression, potentially mediated through its modulation of PTGS2 activity and its interplay with key molecular pathways implicated in disease pathogenesis.

## 5 Conclusions

In this research, we identified the molecular targets of Hairyvein Agrimonia in treating gastric cancer through network pharmacology docking, which were then verified by in vitro cell experiments. Finally, we constructed a survival prediction model for gastric cancer patients using three treatment target genes associated with Hairyvein Agrimonia active components. Our findings provide new targets for researching the anti‐cancer mechanisms of Hairyvein Agrimonia, yet further in‐depth validation and exploration of the specific mechanisms involved are required.

## Author Contributions

Conceptualization: Xiaohui Jin. Methodology: Xiaoyun Ding. Formal analysis: Haizhong Jiang. Investigation: Xuguang Wang. Writing – original draft preparation: Yi Chen. Writing – review and editing: Jiyun Zhu. Supervision and project administration: Hequn He. All authors have read and agreed to the published version of the manuscript.

## Ethics Statement

Ethics approval and consent to participate: AGS and HGC‐27 cells were collected from the Pricella Company (Wuhan, China). The study was conducted per the Declaration of Helsinki. Bioinformatic analysis was obtained from the Ethics Committee of the Ningbo First Hospital.

## Consent

The authors have nothing to report.

## Conflicts of Interest

The authors declare no conflicts of interest.

## Data Availability

The data that support the findings of this study are available from the corresponding author upon reasonable request.
