# Correction to “Unraveling the Anti‐Tumor Effects and Molecular Mechanisms of Hairyvein Agrimonia Herb in Gastric Cancer Through Network Pharmacology and Experimental Validation”

**DOI:** 10.1002/cnr2.70575

**Published:** 2026-05-17

H. He , X. Jin , X. Ding , et al., “Unraveling the Anti‐Tumor Effects and Molecular Mechanisms of Hairyvein Agrimonia Herb in Gastric Cancer Through Network Pharmacology and Experimental Validation,” Cancer Report 8, no. 5 (2025): e70169, 10.1002/cnr2.70169 PMC1208999340391580

In the published article, the affiliation of authors Hequn He, Xuguang Wang, and Yi Chen was incorrect. Their affiliation should read as follows:

“Emergency Department, The First Affiliated Hospital of Ningbo University, Ningbo, China”

The affiliation of author Xiaohui Jin was incorrect. Their affiliation should read as:

“Emergency Department, Haishu District People's Hospital of Ningbo, Ningbo, China”

With the above mentioned changes, the order of affiliations information in the author byline and the affiliations list has been updated as follows:

Hequn He^1^, Xiaohui Jin^2^, Xiaoyun Ding^3^, Haizhong Jiang^3^, Xuguang Wang^1^, Yi Chen^1^, Jiyun Zhu^4^

^1^Emergency Department, The First Affiliated Hospital of Ningbo University, Ningbo, China

^2^Emergency Department, Haishu District People's Hospital of Ningbo, Ningbo, China

^3^Gastroenterology Department, The First Affiliated Hospital of Ningbo University, Ningbo, China

^4^Hepatopancreatobiliary Surgery Department, The First Affiliated Hospital of Ningbo University, Ningbo, China

We apologize for this error.

